# Immune and Circulating Biomarkers in Hepatocellular Carcinoma: Prognostic and Predictive Relevance for Immunotherapy

**DOI:** 10.3390/cancers18213429

**Published:** 2026-10-23

**Authors:** Fady Sidhom, Emin Karabiber, Rafik ElBeblawy, Atakan Babagiray, Timmothy Yacoub, Bassel El-Rayes, Mehmet Akce

**Affiliations:** 1 Taussig Cancer Institute, Cleveland Clinic, Cleveland, OH 44195, USA; 2 Faculty of Medicine, Istanbul Arel University, Büyükçekmece 34010, Türkiye; eminkarabiber22@istanbularel.edu.tr; 3 Department of Medicine, Huntsville Hospital Health System, Decatur Morgan Hospital, Decatur, AL 35601, USA; rafik.elbeblawy@dmhnet.org; 4 Faculty of Medicine, Yeditepe University, Istanbul 34755, Türkiye; atakan.babagiray@std.yeditepe.edu.tr; 5 Internal Medicine, Oregon Health & Science University, Portland, OR 97239, USA; yacoub@ohsu.edu; 6 Division of Hematology and Oncology, UC San Diego Health System, San Diego, CA 92123, USA; 7 Division of Hematology and Oncology, University of Alabama at Birmingham, Birmingham, AL 35233, USA

**Keywords:** hepatocellular carcinoma, immune checkpoint inhibitors, biomarkers, tumor microenvironment, tumor-infiltrating lymphocytes

## Abstract

Hepatocellular carcinoma (HCC) is a leading cause of cancer-related mortality. Immune checkpoint inhibitor (ICI)-based combinations have become the standard first-line systemic therapy, yet objective response rates remain modest (20–36%), and no HCC-specific immune biomarker has been validated to guide selection among standard ICI-based regimens. This review examines the prognostic and exploratory treatment-response associations of potential immune biomarkers in HCC, including markers within the tumor microenvironment (TME) and peripheral blood. A narrative review was conducted evaluating PD-L1 and soluble PD-L1, tumor mutational burden (TMB), tumor-infiltrating lymphocytes (TILs), including machine learning-based assessment, gene expression profiles and tumor-intrinsic pathway alterations, long non-coding RNAs, inflammatory markers (neutrophil-to-lymphocyte ratio, platelet-to-lymphocyte ratio, alpha-fetoprotein), gut microbiota, and liquid biopsy analytes (circulating tumor DNA, circulating tumor cells, cell-free DNA). PD-L1 expression shows prognostic associations and inconsistent associations with ICI outcomes; inter-assay heterogeneity further limits its use, and routine PD-L1 testing is not recommended by current guidelines. TMB is generally low in HCC, with inconsistent associations with ICI response. High CD8^+^ TIL density is associated with improved survival after resection and favorable outcomes in exploratory ICI-treated cohorts, without validated treatment-selective utility. Inflammatory gene signatures and WNT/β-catenin pathway activation are associated with ICI outcomes. Gut microbiome features have shown exploratory associations with anti-PD-1 outcomes, with limited consistency across small datasets. Liquid biopsy markers, particularly ctDNA, show promise for recurrence prediction and treatment monitoring but lack assay standardization. Multiple immune biomarkers demonstrate prognostic value in HCC, and pre-existing immunity characterized by high CD8^+^ T-cell infiltration and inflammatory gene signatures is associated with improved ICI-based therapy outcomes in exploratory studies. However, these exploratory markers have not been validated for routine selection among HCC regimens. Future directions include prospective validation of composite biomarker panels, standardization of assessment methodologies, and integration of artificial intelligence for TME characterization.

## Simple Summary

This review summarizes the growing body of evidence investigating the prognostic and predictive relevance of immune and circulating biomarkers in hepatocellular carcinoma (HCC). Emerging findings from clinical trials, exploratory datasets, and retrospective studies highlight the potential utility of tissue-based immune signatures, molecular components of the tumor microenvironment (TME), circulating inflammatory markers, liquid biopsy and gut microbiome profiles in predicting treatment response and survival outcomes among patients receiving immune checkpoint inhibitors (ICIs). Despite promising findings, substantial challenges remain, including inconsistent study methodologies, limited sample sizes, tumor heterogeneity, and lack of standardized assays. Future large-scale prospective studies, incorporating randomized treatment comparisons and regulated biomarker assessments, are warranted to validate these findings, establish clinically relevant thresholds, and promote biomarker-guided treatment selection for personalized therapeutic strategies in HCC.

## 1. Introduction

Hepatocellular carcinoma (HCC) remains a leading cause of cancer-related mortality worldwide and represents the sixth most common cause of cancer-related death in the United States [1]. The first-line systemic treatment of advanced HCC is now largely centered on immunotherapy-based combinations, replacing tyrosine kinase inhibitors (TKIs). In IMbrave150, atezolizumab, a programmed death-ligand-1 (PD-L1) inhibitor, plus bevacizumab, a vascular endothelial growth factor (VEGF) inhibitor, improved survival over sorafenib and achieved an objective response rate (ORR) of approximately 30% [2]. The HIMALAYA trial showed that durvalumab, a PD-L1 inhibitor, plus tremelimumab, a cytotoxic T-lymphocyte-associated protein 4 (CTLA-4) inhibitor, improved median overall survival (OS) to 16.4 months versus 13.8 months with sorafenib, with ORRs of 20.1% and 5.1%, respectively [3]. More recently, CheckMate-9DW demonstrated a median OS of 23.7 months with nivolumab plus ipilimumab versus 20.6 months with sorafenib or lenvatinib. ORR was also higher with dual immunotherapy, at 36% versus 13% [4]. Collectively, these trials have established ICI-based combinations as preferred first-line options in advanced HCC.

Despite the incorporation of immunotherapy, response rates have remained modest, with only approximately one-third of patients achieving objective response due to primary or acquired resistance to ICIs [5]. Primary resistance is defined as failure to derive meaningful clinical benefit from the onset of ICI therapy, whereas acquired resistance occurs when tumors progress after an initial period of response or disease control [6,7]. Primary resistance is driven by numerous tumor-intrinsic factors such as low tumor antigenicity or neoantigen burden, defects in major histocompatibility complex class I (MHC-I), abnormal tumor cell signaling pathways, tumor-derived exosomes and metabolic reprogramming [8,9,10]. Tumor-extrinsic pathways within the tumor microenvironment (TME) include T cell dysfunction and presence of immunosuppressive cells such as regulatory T-cells (Tregs), myeloid-derived suppressor cells (MDSCs) and tumor-associated macrophages (TAMs) [5,11]. Acquired resistance may involve selection of resistant clones from a heterogeneous tumor population and treatment-associated evolution of somatic alterations, ultimately leading to loss of response to immunotherapy [5].

The primary etiologies that lead to the development of HCC include alcohol-associated liver disease (ALD), metabolic dysfunction-associated steatohepatitis (MASH), viral hepatitis, and other hepatotoxic insults. These conditions can promote chronic hepatic inflammation and contribute to an immunosuppressive TME [12]. HCC TME is a complex and dynamic ecosystem comprising malignant and non-malignant cells, soluble factors, and extracellular matrix components that collectively interact to regulate tumor growth, immune evasion, and therapeutic response [13,14]. It is predominantly composed of immunosuppressive cell populations such as tissue-resident macrophages, TAMs, MDSCs and Tregs, which collectively foster an immune-tolerant environment and suppress effective antitumor immunity [15,16,17].

Several immune characteristics of the HCC TME have emerged as potential biomarkers of ICI efficacy in HCC, including PD-L1 expression, the density and diversity of tumor-infiltrating CD8^+^ T cells and the inflammatory cytokine pathways, such as interferon gamma (IFN-γ) and transforming growth factor beta (TGF-β) [18,19,20]. Additionally, gut microbiota composition is associated with modulation of the ICI therapeutic response, with responders demonstrating greater microbial richness and enrichment of specific bacterial taxa compared with nonresponders [21,22].

**Figure 1:**
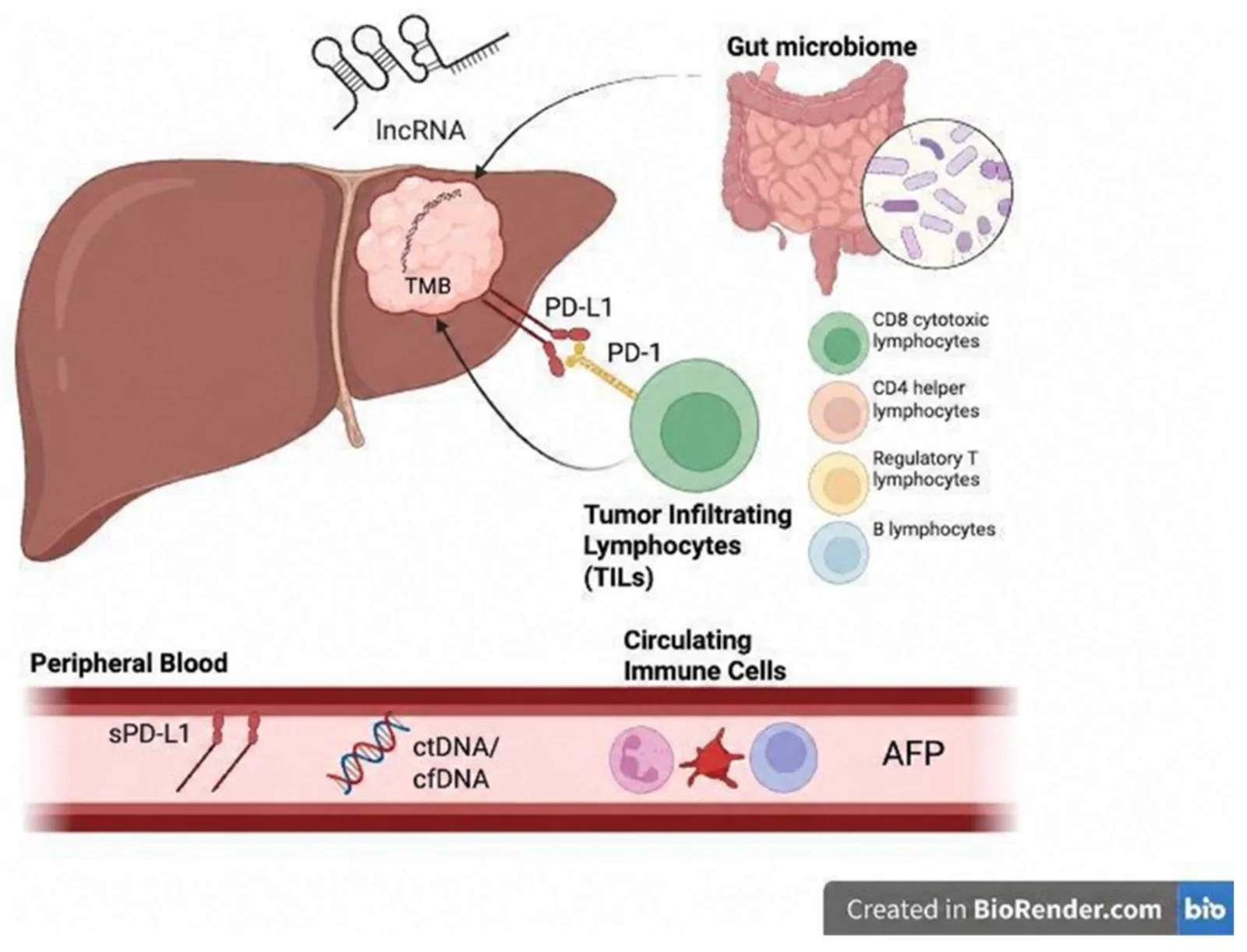
The tumor microenvironment in hepatocellular carcinoma. Created in BioRender. Sidhom, F. (2026) https://BioRender.com/de3ipu5.

As outlined in Figure 1, the key components of the TME include various tumor-infiltrating lymphocyte (TIL) subsets and the PD-1/PD-L1 immune checkpoint axis, which plays a central role in T cell exhaustion. The figure also highlights emerging biomarkers such as long non-coding RNAs (lncRNAs) and tumor-intrinsic molecular features such as tumor mutational burden (TMB). TMB-high states may increase tumor immunogenicity through generation of tumor-specific neoantigens and recruitment or activation of CD8^+^ TILs. The gut microbiome, increasingly recognized as a modulator of hepatic immunity and immunotherapy response, is also depicted. Additionally, peripheral blood biomarkers such as soluble PD-L1 (sPD-L1), circulating tumor DNA (ctDNA) and cell-free DNA (cfDNA) are featured as minimally invasive tools for evaluating disease status and therapeutic response. A deeper understanding of these interconnected elements within the TME is essential for identifying potential predictive biomarkers in optimizing patient selection and advancing the development of novel therapeutics in HCC.

Prognosis in HCC remains heavily influenced by the complex interplay between the TME and the immune landscape. This review explores the prognostic and predictive value of peripheral blood and TME immune markers. Specifically, it focuses on PD-L1, TMB, TILs, gene expression profiles, circulating immune cells, gut microbiota, and liquid biopsy markers including ctDNA, circulating tumor cells (CTCs), and cfDNA. This review was conducted using a narrative approach. Relevant literature was identified primarily through PubMed/MEDLINE and Google Scholar, and publicly available clinical trial registries with a final search date of September 2026. We identified literature of interest using combinations of search frameworks related to the following terms: HCC, ICIs, immunotherapy response and resistance, TME, prognostic biomarkers, predictive biomarkers and circulating or immune biomarkers. Studies were selected based on their relevance to the scope of the review, including clinical trials and biomarker analyses, observational and translational studies, bioinformatic analyses, meta-analyses, and selected ongoing clinical trials. Given the narrative design, a predefined search period, formal inclusion/exclusion criteria, or systematic study-selection protocol was not employed. Throughout this review, prognostic biomarkers are defined as markers associated with the likelihood of a clinical outcome, whereas treatment-predictive biomarkers identify differences in the relative benefit of a specific therapy versus a comparator. Associations within a single treated cohort are described as prognostic or exploratory response associations. Treatment-predictive claims require comparative evidence, preferably supported by a biomarker-by-treatment interaction. Evidence is distinguished by resected disease, perioperative therapy, advanced disease and regimen, or postoperative molecular residual disease; findings are not assumed to transfer between these settings.

## 2. Potential Immune and Circulating Biomarkers in HCC

### 2.1. PD-L1

PD-L1 is an immune checkpoint ligand that plays an important role in regulating immune responses by binding to the PD-1 receptor that is expressed on T cells and various immune cells [23]. PD-L1 is among the earliest biomarkers investigated in HCC and may serve as a prognostic biomarker, but its predictive value remains controversial. A study of 217 patients with HCC found that 17% of tumor cells expressed PD-L1, and PD-L1 expression in both neoplastic and inflammatory cells was significantly associated with tumor aggressiveness as defined by high serum alpha-fetoprotein (AFP), satellite nodules, and macro- and microvascular invasion [24]. In a large genomic profiling study of 1306 hepatic malignancy samples submitted to Caris Life Sciences, including 1152 HCC biopsy specimens, PD-L1 expression was high in 6.1% and low in 11.6% of cases [25]. Notably, PD-L1 expression did not affect the duration of ICI treatment.

#### 2.1.1. Advanced HCC Treated with ICI Monotherapy

Exploratory and correlative analyses of data subsets in advanced HCC investigated PD-L1 associations with treatment outcomes among patients treated with ICIs. Analysis of fresh and archival tissue samples from the dose-escalation and dose-expansion phases of the CheckMate 040 trial showed that patients receiving nivolumab had higher response rates and longer OS in the PD-L1–positive subgroup (defined as ≥1% expression) compared to the PD-L1–negative subgroup; however, complete (CR) and partial responses (PR) were also observed among patients with negative PD-L1; median OS of 28.1 months versus 16.6 months respectively [26]. KEYNOTE-224 evaluated single-agent pembrolizumab in advanced HCC and presented two distinctive correlative analyses: combined positive score (CPS) and tumor proportion score (TPS) reflecting PD-L1 as a potential prognostic biomarker. CPS was defined as the number of PD-L1–positive cells (tumor cells, lymphocytes, and macrophages) divided by the total number of viable tumor cells, multiplied by 100. TPS was defined as the percentage of viable tumor cells with partial or complete membrane staining of PD-L1 (≥1%) relative to all viable tumor cells in the sample. CPS was associated with better ORR but inconsistent with TPS, likely due to substantial inter-assay heterogeneity in the detection of PD-L1 expression [27]. Both studies evaluated advanced HCC treated with ICI monotherapy. Their single-arm biomarker analyses cannot establish differential treatment benefit. Pinato and colleagues evaluated 100 HCC specimens tested with 5 different PD-L1 immunohistochemistry (IHC) assays. They found significant inter-assay variability in the proportion and intensity of PD-L1 expression in immune cell infiltrates [12]. The inter-assay heterogeneity was found not only in malignant cells but also in TILs and non-tumorous cirrhotic tissues.

#### 2.1.2. Advanced HCC Treated with Atezolizumab-Based Regimen

In an exploratory molecular analysis of tumor samples from 358 patients with HCC enrolled in GO30140 phase 1b or IMbrave150 phase 3 trials, treated with atezolizumab plus bevacizumab, atezolizumab monotherapy or sorafenib, there appeared to be a trend of increasing response rates with increasing PD-L1 immune cell or tumor cell scores. This did not reach statistical significance and was not consistent across the data [20].

#### 2.1.3. Resectable HCC Treated with Perioperative ICI

In a correlative analysis of the phase II trial—NCT03222076—LaPelusa et al. evaluated PD-1 and PD-L1 status and outcomes in the perioperative setting using neoadjuvant nivolumab plus ipilimumab or nivolumab monotherapy [28]. Patients with resectable HCC were included and serial tissue biopsies were obtained prior to and after completion of ICIs and after surgical resection. Patients who achieved major pathological response (MPR) (>70% tumor necrosis) had a greater increase in intratumoral expression levels of PD-1 (20.17% versus 0.40%; *p* = 0.048), while not consistent with PD-L1 (7.69% versus 0.57%; *p* = 0.26) [28].

Based on these inconsistent results with baseline PD-L1 testing, routine testing for PD-L1 status is not recommended in HCC by national guidelines [29]. Furthermore, systemic therapy approvals for ICI-based regimens were granted without selection for PD-L1 status or testing for this biomarker. The cited exploratory studies have not established a PD-L1 threshold for selecting ICI-based regimens in HCC. Future directions include evaluating PD-L1 expression on CTCs, where a recent study found that high PD-L1 CTC burden was associated with worse PFS and OS [30].

### 2.2. Soluble PD-L1/PD-1

Soluble PD-L1 (sPD-L1) and soluble PD-1 (sPD-1) are circulating forms of the PD-L1 ligand and PD-1 receptor measured in peripheral blood, distinct from their membrane-bound counterparts [31]. A meta-analysis that included 11 studies encompassing over 1200 patients with HCC who received various locoregional therapies found that high sPD-L1 (defined by various cutoffs; ranged from 0.0013 to 11,200 ng/mL) was associated with worse OS (HR = 2.46, 95% CI 1.74–3.49, *p* < 0.001) [32]. Similar findings were observed for disease-free survival (DFS), relapse-free survival (RFS), time to progression (TTP), and tumor-free survival (TFS). In contrast, sPD-1 showed no significant association with OS (HR = 1.19, 95% CI 0.55–2.56, *p* = 0.657), nor with DFS or TFS [32]. Lack of standardized reference levels for sPD-L1 is one of the key limitations and future studies are needed to further evaluate the role of sPD-L1 and sPD-1 as prognostic or exploratory response-associated biomarkers in HCC [33].

### 2.3. Tumor Mutational Burden

TMB refers to the total number of somatic mutations present within the tumor genome, expressed as mutations per megabase (mut/Mb) of DNA analyzed. TMB is viewed as a potential pan-tumor biomarker, but HCC has a lower median TMB of 4 mut/Mb relative to other solid tumor types [34,35]. A study evaluated the relationship between TMB and ORR across 27 tumor types treated with anti-PD-1 or anti-PD-L1 therapy and found a strong linear correlation between ORR and the median number of coding somatic mut/Mb, including HCC [35].

Another study analyzing an HCC cohort from the Cancer Genome Atlas (TCGA) classified 364 patients with HCC into low- and high-TMB groups. High TMB was associated with worse prognosis compared to low TMB (*p* = 0.017) [36]. This study defined TMB-high as the top 20% of the cohort, whereas another study used a cutoff of 4.9 mut/Mb, highlighting the lack of a standardized HCC-specific threshold [37]. Also of note, the TCGA study found that follicular helper cells and activated natural killer cells were enriched in TMB-high patients while resting dendritic cells were enriched in TMB-low patients, which further characterizes the TME in HCC.

#### 2.3.1. Advanced HCC Treated with Atezolizumab-Based Regimens

Zhu and colleagues further explored the association between TMB and response rates by evaluating HCC baseline patient-matched tumor-blood samples from both phase 1b GO30140 and the phase 3 IMbrave150 studies evaluating atezolizumab (anti-PD-L1) with or without bevacizumab (anti-VEGF) in advanced unresectable HCC [20]. Median TMB was assessed using whole-exome sequencing (5.6 mut/Mb) and FoundationOne (4.4 mut/Mb), and patients were subsequently categorized into tertiles: TMB-low, TMB-medium, and TMB-high. Higher ORR among the TMB-high group versus the TMB-low group (56% vs. 35% respectively) was revealed in the GO30140 cohort. Nevertheless, a similar result was not validated in the IMbrave 150 cohort; ORR was 37% in the TMB-high group and 38% in the TMB-low group. In the phase 1b GO30140 cohort, they also found a trend towards PFS benefit in the TMB-high group, but this was not statistically significant (HR 0.63, *p* = 0.10). Overall, this highlights an unvalidated and inconsistent association between TMB and outcomes in advanced HCC treated with atezolizumab-based regimens. Assay methods and thresholds require standardization before treatment-selective utility can be assessed.

#### 2.3.2. Localized HCC Treated with Surgery

In the postoperative surveillance setting, ctDNA-based TMB measured using Guardant360 has shown an association with HCC recurrence [38]. These assays have shown correlations with NAFLD, as a cirrhosis etiology, and human telomerase reverse transcriptase (hTERT)/TP53 mutations as oncogenic drivers [39].

#### 2.3.3. Advanced HCC Treated with Targeted Therapy and Immunotherapy

A retrospective study in Wuhan screened 151 patients, of whom 127 were included in the analysis with unresectable advanced HCC receiving targeted therapy and immunotherapy to implement next-generation sequencing (NGS) for exploratory prognostic and treatment-response associations [40]. Baseline blood samples underwent 680-gene NGS and PD-L1 expression was assessed by IHC [40]. Using this data, the investigators developed a prognostic index model incorporating TP53, TERT, PD-L1, and TMB to estimate outcomes within the treated cohort. Patients with TMB of ≥5 mut/Mb achieved prolonged median OS of 59 months versus 41 months with TMB < 5 mut/Mb, *p* = 0.001 [40]. Notably, a TMB of ≥5 mut/Mb was an independent prognostic biomarker for OS in the multivariable model (HR 86.67, 95% CI 4.00–1876.48, *p* = 0.004), though the extremely wide confidence interval indicates substantial imprecision. An ongoing phase 1/2 trial is evaluating STAR0602, a bifunctional antibody-fusion molecule, in patients with antigen-rich advanced solid tumors, including those with TMB-High status, with an estimated completion date in late 2026 [41].

### 2.4. Tumor-Infiltrating Immune Cells

Tumor-infiltrating immune cells include T lymphocytes, macrophages, dendritic cells, and natural killer cells. TILs specifically refer to the lymphocytic component, which reflects the host immune response to the tumor [42]. In HCC, the immune microenvironment plays a critical role in tumor progression and response to therapy, and the composition of TILs has been associated with both clinical outcomes and response to immunotherapies, supporting their role as prognostic and exploratory response-associated biomarkers [43].

#### 2.4.1. Prognostic Biomarker Post-Surgical Resection

Among patients who underwent surgical resection, correlative analyses of CD8^+^ TILs, as a potential prognostic biomarker, have been studied in HCC. In a meta-analysis of 3509 patients across 21 retrospective studies from seven countries and all tumor stages, Xu et al. found that high intratumoral CD8^+^ TIL density in surgical specimens was associated with improved OS (HR = 0.676, *p* = 0.001) and DFS (HR = 0.712, *p* = 0.002) [44]. High CD8^+^ TIL density at the tumor margin was also associated with improved OS (HR = 0.577, *p* < 0.001) [44]. Although the included studies had varying features in immune cell location, staining methods, and cut-off definitions, these findings support the prognostic potential of CD8^+^ TILs in HCC, limited by retrospective data. Gao et al. similarly examined the prognostic value of TIL density in resected HCC specimens using a two-phase study design that included treatment-naïve patients with histologically confirmed primary HCC after hepatectomy [45]. The study included a discovery cohort of 315 patients and a separate validation cohort of 343 patients. TIL density was categorized as high (TILs ≥ 50%), intermediate (10% ≤ TILs < 50%), and low (TILs < 10%). Both the high and intermediate TIL groups had better OS and DFS compared to the low-TIL group, but this study was limited by only 11% of patients having high TIL density, which may limit its generalizability.

#### 2.4.2. Advanced HCC Treated with ICI Monotherapy

In the setting of ICI therapy, a biomarker analysis of fresh and archival tumor samples from CheckMate 040, researchers evaluated patients treated specifically with nivolumab monotherapy during the dose-escalation and dose-expansion phases [26]. The investigators found that increased CD3^+^ and CD8^+^ TILs in baseline samples showed a trend towards improved OS, although this was non-significant (*p* = 0.08). This analysis was also limited by a modest enrollment size for the biomarker evaluation, which included 195 patients with assessable samples. Future studies evaluating TILs in pre-treatment and on-treatment tumor samples are needed to better characterize the potential prognostic-associated role of CD3^+^ and CD8^+^ TILs.

#### 2.4.3. Advanced HCC Treated with Atezolizumab Plus Bevacizumab

Zhu and colleagues molecularly analyzed tumor samples from the GO30140 and IMbrave150 studies among patients treated with atezolizumab plus bevacizumab to investigate associations between TILs and treatment outcomes [20]. In the GO30140 cohort of 61 tumor samples, a multiplex IHC analysis showed that responders with a complete or partial response had a higher density of CD8^+^ T cells, CD3^+^ T cells and GZMB^+^CD3^+^ T cells than non-responders with stable (SD) or progressive disease (PD). Similar results were seen in their IMbrave150 cohort of 177 samples with a higher density of CD8^+^ T cells in responders. Additionally, the multiplex IHC analysis revealed that responders showed higher expression of MHC class 1 on the surface of tumor cells than nonresponders.

#### 2.4.4. Advanced HCC Treated with Anti-CTLA-4 Antibody Plus Ablation

TIL dynamics have also been evaluated in studies of CTLA-4-directed therapy. In a 2017 study, Duffy and colleagues measured the amount of activated T cells both at baseline and following tremelimumab combined with ablation among patients with advanced HCC [46]. While 32 patients were enrolled and treated with tremelimumab, the intratumoral immune analysis was conducted on a subset of 12 evaluable specimens. In this subset, they found that both CD4^+^ and CD8^+^ T cells were increased following immunotherapy; specifically, CD8^+^ T cells increased by 2-fold over baseline, and this was sustained for at least 12 weeks. Responders had higher mean positive pixel count (mPPC) percentages for CD3^+^ and CD8^+^ T cells (10.21% and 8.1%, respectively) compared to non-responders (2.44% and 2.00%, respectively) [46]. Although this was a small cohort of only 12 patients, it does provide valuable insight into the immunotherapeutic effect on TME.

#### 2.4.5. Resectable HCC Treated with Perioperative ICI Therapy

A subsequent study evaluated TILs and outcomes during anti-PD-1 therapy with or without CTLA-4 blockade in the perioperative setting [28]. In a phase II trial, neoadjuvant nivolumab plus ipilimumab or nivolumab monotherapy was evaluated in 18 patients with resectable HCC. Patients achieving MPR demonstrated significantly greater increases in intratumoral CD8^+^ expression compared to non-responders (26.92% vs. −0.04%; *p* = 0.026) [28]. This proof-of-concept trial identified treatment-related immune changes associated with pathological responses. Yet their potential treatment-associated response is limited by small sample size, and such changes are exploratory pharmacodynamics rather than validated pretreatment selection markers.

TILs could have potential prognostic utility in HCC. However, transitioning TILs from observational data into routine clinical practice requires overcoming the limitations of small sample sizes and heterogeneous retrospective study designs. Future research must prioritize large prospective trials with uniform scoring methodologies and evaluation of biopsies before and after treatment for further validation of TILs as a promising biomarker in HCC.

#### 2.4.6. Machine Learning and TILs

Machine learning-based TIL assessment refers to the use of computational algorithms such as artificial intelligence (AI) to measure immune cell infiltration and evaluate the arrangement of lymphocytes within tumor tissue. In HCC, this tool may help overcome the limitations of conventional TIL assessment, including observer variability and heterogeneous scoring methods, to improve consistency and TIL quantification as a digital biomarker.

##### Prognostic Biomarker Post-Surgical Resection

Chen and colleagues developed an AI-based CD8^+^ TIL scoring system called the Automated CD8^+^ Tumor-Infiltrating Lymphocyte Scoring system (ATLS-8), which uses deep neural network and random forest models to identify tumor regions and measure CD8^+^ TIL density in H&E and CD8-stained whole-slide images [47]. In this multicenter retrospective study of 514 patients who underwent liver resection, patients with high ATLS-8 scores had better 5-year OS than those with low ATLS-8 scores in the discovery cohort (65.03% vs. 34.05%; HR, 2.40; 95% CI, 1.37–4.19; *p* = 0.015), validated in two independent cohorts.

A retrospective study by Zhang et al. evaluated 353 patients with early-stage HCC who underwent curative-intent surgical resection [48]. The researchers used an artificial neural network algorithm to identify a metric called TIL densityK50, which uses the density of each cell based on its distance to the 50 nearest lymphocytes. That metric was associated with improved DFS in univariate (hazard ratio, 0.282; 95% CI, 0.181–0.439; *p* < 0.0001) and multivariate analysis (hazard ratio, 0.315; 95% CI, 0.115–0.860; *p* = 0.024) [48]. TIL density and the spatial relationship with other immune cells provide a basis for exploring AI-derived prognostic metrics and recurrence-risk assessment in resected HCC.

##### Advanced HCC Treated with Atezolizumab Plus Bevacizumab or Anti-PD-1 Monotherapy

Machine learning-based TIL assessment has also been evaluated in the immunotherapy setting. Scheiner et al. used a supervised machine learning algorithm to measure immune cell infiltration on 102 pretreatment samples in BCLC stage C patients treated with atezolizumab and bevacizumab [49]. Patients were separated into ICI-low and ICI-high groups using a cutoff of ≥236 ICIs/mm^2^, with the ICI-high group demonstrating significantly improved OS compared to the ICI-low group (20.9 months [95% CI: 13.8–27.9] vs. 15.3 months [95% CI: 6.0–24.6]; *p* = 0.026). Another study conducted by Kim et al. used an AI model to measure spatial distribution of immune and non-immune cells and found that high tumor endothelial cell (TEC) density was associated with longer PFS (HR 0.51 [0.27–0.97]; *p* = 0.037) in an atezolizumab plus bevacizumab cohort but not in an anti-PD-1 monotherapy cohort (HR 1.02 [0.59–1.77]; *p*  =  0.935) [50]. Conversely, the same study found that high intratumoral TIL densities were associated with longer PFS in the anti-PD-1 monotherapy cohort (HR 0.50 [0.25–0.99]; *p* = 0.042) but not in the atezolizumab plus bevacizumab cohort (HR 0.92 [0.50–1.69]; *p* = 0.762).

Current data suggest that AI-derived TIL metrics may provide prognostic information in resected HCC and show exploratory associations with outcomes in advanced HCC treated with different immunotherapy regimens. However, these studies remain largely retrospective and observational. Also, differences in algorithms, tissue processing, staining methods, and cutoff values may limit immediate clinical implementation. Future directions should include validation in larger prospective cohorts, standardization of algorithms and tissue processing methods, and development of biomarker-based models that can support treatment selection in HCC.

### 2.5. Gene Expression and Alterations

#### 2.5.1. Gene Expression Profiles

Gene expression profiles (GEPs) measure RNA expression across multiple genes, typically using sequencing, to identify patterns termed gene expression signatures [26]. In exploratory analyses, GEPs in HCC have demonstrated prognostic value. However, their definitive predictive value remains investigational. Routine molecular profiling for HCC patients is not supported by national guidelines [29].

#### 2.5.2. Advanced HCC Treated with Nivolumab

An exploratory analysis of 195 samples from the dose-escalation and dose-expansion phases of CheckMate 040, in which patients received nivolumab, evaluated various gene expression signatures and revealed that an inflammatory signature consisting of CD274 (PD-L1 messenger RNA), CD8A, LAG3, and STAT1 was associated with improved overall response rate (*p* = 0.05) and OS (*p* = 0.01) [26].

#### 2.5.3. Advanced HCC Treated with Atezolizumab Plus Bevacizumab

Molecular correlates in patients with advanced HCC treated with atezolizumab in combination with bevacizumab also showed that pre-existing immunity (high expression of CD274, T-effector signature and intratumoral CD8^+^ T cell density) was associated with better clinical outcomes. Conversely, the researchers utilized RNA-seq and gene set enrichment analysis to measure biological pathways, revealing that the upregulation of angiogenesis, cholesterol homeostasis, bile acid metabolism and Notch signaling pathways was all associated with lack of response [20]. The same study also evaluated an atezolizumab plus bevacizumab response signature (ABRS) composed of the top ten genes from a genome-wide differentially expressed gene (DEG) analysis. It used the median score to stratify patients into high or low ABRS. The ABRS high group had a PFS benefit (*p* = 0.013) when compared to the ABRS low group, with similar results for CD274 high and T-effector high groups [20]. These exploratory analyses suggest that signatures may be regimen-dependent, but comparisons of outcomes between biomarker-high and biomarker-low patients within one treatment arm do not establish treatment-selective utility.

Existing GEP signatures have been largely derived from studies including advanced HCC treated with nivolumab monotherapy or atezolizumab/bevacizumab combination. In future studies, validation should distinguish disease stage, treatment regimen, specimen timing, and whether the analysis included a treatment comparator and biomarker-by-treatment interaction to improve the applicability of GEP across the full disease spectrum.

### 2.6. Tumor-Intrinsic Pathways

Tumor-intrinsic pathways refer to the internal cellular signaling cascades and genetic drivers that regulate cancer cell growth, survival, and immune evasion. In solid tumors like HCC, extensive study of the genetic profile has identified recurrent somatic oncogenic drivers and tumor suppressors within specific cellular pathways, such as WNT signaling, TP53, PI3K-mTOR, and MAPK. Such pathways not only mediate oncogenesis but also influence the TME and response to systemic therapies. Specific gene alterations could also be associated with responses to ICIs.

#### Advanced HCC Treated with Immunotherapy

In a prospective cohort of 127 patients with advanced HCC, Harding et al. utilized clinical NGS to evaluate molecular pathway alterations and their correlation with outcomes in patients treated with single-agent or dual ICIs [51]. They found that activated WNT/beta-catenin signaling was associated with a lower disease control rate (DCR) (0% in WNT/β-catenin-altered versus 53% in unaltered tumors within the ICI-treated subgroup), as well as shorter PFS and median OS. The same study also reported that TP53 alterations are associated with less CD8^+^ T-cell infiltration, resulting in the downregulation of the immune response. By including multiple ICIs with different mechanisms of action, including anti-PD-1/PD-L1, CTLA-4, KIR, and LAG-3 agents, this small heterogeneous subgroup provides exploratory evidence of an association with ICI outcomes.

Within the IMbrave150 and GO30140 cohorts, two specific gene alterations—CTNNB1 wild-type and TERT mutant tumors—were associated with greater benefit with atezolizumab and bevacizumab compared to sorafenib. The limited sample size of the correlative analysis warrants further validation [20]. Notably, the ongoing Phase I/II SAPHIRE trial (NCT06811116) is evaluating the combination of the mTOR inhibitor sapanisertib with the TKI cabozantinib in β-catenin-mutated tumors, representing an active clinical trial directly assessing the therapeutic implications of a tumor-intrinsic genomic alteration [52].

### 2.7. Long Non-Coding RNAs

lncRNAs are non-coding transcripts longer than 200 nucleotides that can regulate gene expression [53]. lncRNAs in HCC have shown promising prognostic value, as their expression has been linked to tumor size, cancer stage and survival outcomes [54]. A bioinformatics study by Peng et al., across 33 cancer types, investigated the role of the lncRNA MIR155HG (MIR155 host gene) as a prognostic biomarker and its association with immune infiltration and immune checkpoint molecule expression [55]. They found that high expression of the lncRNAs in the MIR155 host gene was significantly correlated with infiltrating immune cells as well as with PD-L1 and CTLA4, supporting a hypothesis concerning tumor immunity across multiple cancer types, including HCC. Although this study was across tumor types, larger studies evaluating MIR155 specifically within HCC are needed.

#### HCC-Specific lncRNAs

Zhang et al. conducted a retrospective multi-cohort bioinformatics study in which they identified and validated a prognostic signature comprising 10 immune-related lncRNAs in HCC. Using a TCGA training cohort (*n* = 368) and an independent external validation cohort from GEO (GSE76427, *n* = 115), the signature stratified patients into high- and low-risk groups [56]. They found that the immune-related signature was correlated with disease progression and worse survival. These retrospective genomic datasets support potential prognostic utility. Future prospective ICI-based cohorts are needed to assess treatment response predictive value.

In a more recent study, Deng et al. performed a comprehensive bioinformatics analysis of mRNA sequencing data and lncRNA expression profiles from 346 HCC tumor specimens and 50 adjacent normal tissue samples in the TCGA database to identify lncRNAs associated with HCC oncogenesis [57]. They identified five hub lncRNAs (AC091057, AC099850, AC012073, DDX11-AS1, and AL035461) associated with inferior OS in samples with high expression profiles. The authors caution that these findings were based on a subset of the TCGA database and require further validation.

The cited lncRNA evidence remains predominantly bioinformatic rather than prospectively validated in ICI-treated HCC. Future directions include standardizing detection protocols, conducting large-scale prospective validation studies and ultimately developing lncRNA signatures to predict immunotherapy response [58].

### 2.8. Inflammatory and Tumor Markers

In the TME, inflammation promotes tumor growth, angiogenesis, and metastasis while weakening anti-tumor immunity [59]. This underlying inflammatory state causes measurable changes in peripheral white blood cells and platelets, serving as a key indicator of the relationship between the TME and the systemic immune system. Additionally, tumor markers such as AFP, which is produced primarily by hepatic tissue and the visceral endoderm of the yolk sac, are predominantly elevated in pathological conditions such as HCC [60]. Recent evidence highlights the joint role of these tumor markers and systemic inflammatory parameters, including the neutrophil-to-lymphocyte ratio (NLR) and platelet-to-lymphocyte ratio (PLR), in association with treatment outcomes and prognosis [61,62].

#### Unresectable HCC Treated with Atezolizumab and Bevacizumab

Studies show that inflammatory parameters offer vital prognostic value across various HCC therapies, including unresectable HCC treated with atezolizumab and bevacizumab, where patients with NLR ≥ 5 and PLR ≥ 300 had shorter OS, and advanced HCC treated with nivolumab, where both pre- and post-treatment NLR and PLR tertiles were strongly associated with survival outcomes [63,64]. Despite the noticeable contribution of the inflammatory marker within the TME, their levels are confounded by other underlying liver diseases and are not entirely tumor-specific. NLR may be affected by systemic infection, hepatic inflammation, and cirrhosis severity [65,66]. PLR may be confounded by thrombocytopenia related to portal hypertension, hypersplenism, and advanced fibrosis [67,68]. AFP may also increase with active hepatitis, hepatic regeneration, fibrosis, and cirrhosis independent of HCC [69,70].

### 2.9. Inflammatory Markers

NLR, PLR, and AFP have shown prognostic or response-associated relationships in advanced HCC treated with nivolumab or sorafenib [64,71,72,73]. A meta-analysis in 2017 of 24 studies (*n* = 6318) evaluated patients with HCC across heterogeneous settings, including surgical resection, transplantation, transarterial chemoembolization (TACE), radiofrequency ablation, and systemic sorafenib, suggested that both NLR and PLR are potential prognostic biomarkers for clinical outcomes in this population [74]. Notably, because this meta-analysis was conducted in 2017, it did not include patients treated with immunotherapy-based regimens, which have since become the standard of care for advanced HCC.

#### 2.9.1. Advanced HCC Treated with Immunotherapy

The CheckMate 040 study further evaluated these biomarkers to provide informative data regarding NLR, PLR and AFP levels as measurement tools of the inflammatory response within the TME. The study reported a median NLR of 2.87, with a nonsignificant trend towards higher NLR among patients with progressive disease compared with treatment responders (*p* = 0.31). Patients in the lowest NLR tertile demonstrated significantly improved OS compared with those in the intermediate or highest tertiles (*p* = 0.015) [26]. Similarly, the PLR was significantly lower among treatment responders than non-responders (*p* = 0.05). Lower PLR levels were also strongly associated with improved OS compared with higher PLR levels (*p* = 1.38 × 10^−7^) [26]. In comparison, IMbrave150 did not directly evaluate NLR or PLR as prespecified biomarkers; however, it evaluated AFP as a stratification factor, which will be outlined in the following section.

Retrospective studies and correlative analyses of prospective trials suggest a potential prognostic role for circulating inflammatory markers; however, prospective validation is needed to confirm these findings.

#### 2.9.2. Alpha-Fetoprotein

AFP is a glycoprotein that serves an immunoregulatory role during fetal development. It is a member of the albumin-like protein family, which also shares structural and functional similarities with other plasma proteins, including human serum albumin and vitamin D–binding protein [75]. It represents one of the most widely used biomarkers for HCC diagnosis and can also be produced by other tumors, including germ cell tumors and hepatoblastoma. AFP levels are normally low in adults but may increase with hepatic regeneration, hepatitis, or cancer when synthesized above certain levels. AFP synthesis within tumors is more common among tumors characterized by large size, poor differentiation, and vascular invasion. Consequently, serum AFP levels serve as a critical determinant of prognosis in patients with HCC [76].

#### 2.9.3. Advanced HCC Treated with Immunotherapy

In the Checkmate 040 study, OS was numerically higher among patients with low AFP levels (<400 μg/L; *n* = 92) at 16.8 months (95% CI, 13.3–20.2) compared with 13.0 months in patients with high AFP levels (≥400 μg/L; *n* = 57) [26]. Although slight differences were observed in the 24-month OS, there were no substantial differences in the ORR and DCR [26]. Clinical evidence from the IMbrave150 trial demonstrates that decreasing AFP levels following therapy are closely associated with improved treatment outcomes [77].

#### 2.9.4. GALAD/BALAD-2 Scores as Biomarkers for Treatment Efficacy

The National Liver Cancer Screening Trial (TRACER)—NCT06084234—is currently evaluating whether the GALAD score [A multi-parameter formula incorporating gender, age, lens culinaris agglutinin-reactive AFP (AFP-L3), AFP, and des-gamma-carboxy prothrombin (DCP)] outperforms traditional ultrasound-based screening [78].

In the future, the clinical utility of the GALAD score could be effectively shifted from a diagnostic framework to a dynamic tool for monitoring therapeutic efficacy. In a retrospective study involving 220 patients with HCC undergoing either transarterial or predominantly sorafenib-based systemic treatments, baseline scoring systems GALAD and BALAD-2 [Bilirubin, Albumin, AFP, AFP-L3, and DCP] were associated with 3-month treatment response evaluated via mRECIST criteria [79]. Specifically, among responders to transarterial treatment, baseline GALAD scores were significantly lower compared to patients with refractory disease (0.97 versus 5.32; *p*  <  0.001) and demonstrated prognostic discrimination for OS (AUC = 0.715, *p* < 0.001), outperforming individual standalone biomarkers including AFP, AFP-L3, and DCP [79]. Among responders to systemic treatment, the median GALAD score at baseline was similar to non-responders at three months (3.68 versus 4.77; *p*  =  0.133); however, within the BCLC-B subgroup, GALAD score was significantly lower in responders versus non-responders (*p* = 0.05) [79]. The median BALAD-2 score showed a significant association with systemic treatment response at three months (3.55 versus 10.08; *p* < 0.001) [79]. Neither GALAD nor BALAD-2 scores were associated with OS among patients treated with systemic therapy [79]. These findings suggest that these biomarker-based scoring systems hold substantial promise as non-invasive prognostic tools in HCC; however, they do not establish GALAD or BALAD-2 as markers for ICI selection or justify biomarker-guided changes in treatment.

### 2.10. Gut Microbiota

#### 2.10.1. The Gut Liver Axis and Hepatocarcinogenesis

The microbial ecosystem in the human gut maintains extensive and critical interactions with the host immune system. This complex crosstalk is essential for the development of a diverse pool of immune cells and the maintenance of gut homeostasis, which creates a favorable environment for commensal bacteria [80].

In the context of chronic liver disease, several molecular pathways link the gut microbiome to hepatic pathology. Pathological changes in the intestinal barrier increase permeability, facilitating the translocation of microbiota-associated molecular patterns (MAMPs), such as lipopolysaccharide (LPS), through the portal vein into the liver. Once in the liver, these MAMPs activate resident cells, including Kupffer cells and hepatic stellate cells (HSCs). For instance, LPS activates Toll-like receptor 4 (TLR4), triggering tumor-promoting inflammation and inducing HSCs to secrete epiregulin, a potent hepatomitogen that drives the development of HCC. Furthermore, dysbiosis-induced alterations in metabolic pathways lead to the accumulation of secondary bile acids, such as deoxycholic acid (DCA). These metabolites can increase DNA damage and activate the mTOR pathway in hepatocytes, further accelerating tumor growth [81].

Interpretation of microbiome signatures in HCC, however, is complicated by several potential confounders that may limit reproducibility across studies. Geographic origin can substantially influence gut microbial composition and may even obscure microbiome differences attributable to the underlying liver-disease etiology [82]. Dietary patterns are another major determinant of gut microbial composition, with highly individualized microbial responses to specific foods and dietary exposures [83]. Medication exposure should also be considered, particularly antibiotics, which can markedly disrupt microbial composition and function in patients with cirrhosis [84], and proton-pump inhibitors, which have been associated with significant shifts in gut microbial composition and metabolic function [85]. Furthermore, cirrhosis itself contributes substantially to gut dysbiosis in patients with HCC [86], while the underlying liver-disease etiology and disease stage, including viral hepatitis, ALD, and MASH, may further shape microbial profiles [82,87]. Collectively, these sources of biological and environmental heterogeneity should be carefully accounted for when comparing cohorts and validating microbiome-based prognostic or predictive biomarkers in HCC.

The significant heterogeneity of inter-individual microbial compositions in response to ICIs suggests that targeting specific gut microbes could augment checkpoint blockade therapies in HCC [88]. Small observational HCC studies report associations between microbiome composition and outcomes during anti-PD-1 therapy. Metagenomic analysis of fecal samples shows that responders exhibit significantly higher species richness and a greater count of microbial genes compared to non-responders [21]. Notably, dissimilarities between these groups become observable as early as six weeks into treatment. In non-responders, Proteobacteria typically increases by the third week and often becomes the dominant phylum by week 12. Conversely, specific bacterial species act as “immunotherapy probiotics” by enhancing anti-tumor immunity. Research has identified approximately 20 such species, most notably *Akkermansia muciniphila* and *Ruminococcaceae* spp. [21].

In non-HCC cancer studies, fecal microbiota transplantation (FMT) from ICI responders into germ-free mice has been shown to restore anti-PD-1 therapeutic efficacy, an effect largely attributed to the commensal bacterium *Akkermansia muciniphila* [89]. Mechanistically, oral supplementation with *A. muciniphila* actively orchestrates a robust antitumor immune response through an interleukin-12 (IL-12)-dependent pathway, which activates dendritic cells, stimulates T cells, and upregulates interferon-gamma production [89]. This cytokine cascade subsequently enhances the intratumoral recruitment of effector CXCR3^+^ CD4^+^ T and CD8^+^ T lymphocytes [89]. These exploratory findings highlight the potential immunomodulatory role of gut microbiota in cancer immunotherapy and present them as potential prognostic and treatment-associated biomarkers. Future prospective and randomized trials are needed to validate their potential to overcome therapeutic resistance and improve clinical outcomes. NCT05273255—a pan-malignancy trial—is now investigating FMT in malignancies including HCC, evaluating whether transferring the microbiome from an ICI ‘responder’ to a ‘non-responder’ may rescue patients who have progressed on standard immunotherapy [90].

#### 2.10.2. Microbial Signatures and Survival Outcomes

In a mixed hepatobiliary cohort, metagenomic sequencing of fresh stool samples was analyzed as microbial signatures to evaluate their potential association with prognosis in patients with advanced hepatobiliary cancers and HCC treated with immunotherapy [91]. In that prospective study, 30 patients with HCC and 35 patients with biliary tract cancer receiving anti-PD-1-based ICI were enrolled, and fecal microbial signatures collected before and after ICIs were analyzed. Specific taxonomic enrichment patterns were significantly associated with better OS and PFS. High abundance of *Lachnospiraceae bacterium* GAM79 was associated with a median OS that was not reached, compared with 13.8 months (*p* = 0.023), and a median PFS of 7.9 vs. 5.1 months (*p* = 0.020). Similarly, high abundance of *Alistipes* sp. Marseille-P5997 was associated with a median OS that was not reached, compared with 13.8 months (*p* = 0.021), and a median PFS of 9.0 months vs. 5.2 months (*p* = 0.013) [91]. Conversely, enrichment of *Veillonellaceae* in the non-clinical benefit group was associated with shorter survival and more rapid disease progression [91]. Whether the gut microbiome can be leveraged as a non-invasive prognostic or response-monitoring tool to optimize anti-PD-1 therapy and inform individualized treatment strategies in HCC raises an ongoing research question. These estimates arise from a mixed HCC/biliary tract cancer cohort and should not be interpreted as HCC-specific treatment-predictive effects.

#### 2.10.3. Advanced HCC Treated with Second- or Third-Line Nivolumab

The gut microbiome of 8 patients with advanced HCC treated with nivolumab as a second- or third-line systemic treatment was collected before starting ICI, at the time of progression, and at objective response at 5–7 months [92]. Responders to nivolumab demonstrated a higher Shannon index, a measure of microbial diversity reflecting both species’ richness and evenness, indicating significantly greater bacterial richness and diversity compared to non-responders (*p* = 0.036) [92]. While the presence of *Akkermansia* species, *Citrobacter freundii*, and *Enterococcus durans* were associated with a favorable response, higher levels of *Escherichia coli*, *Enterococcus faecium*, and *Veillonella* were associated with therapeutic failure [92].

HCC-specific microbiomes could serve as prognostic and potentially treatment-associated biomarkers for immunotherapeutic response; however, current exploratory studies do not establish treatment-selective utility or efficacy of microbiome-directed interventions. Larger prospective studies should control for liver disease, medication exposure, diet, and geography and include independent validation and appropriate treatment comparisons.

### 2.11. Liquid Biopsy

Liquid biopsy represents a minimally invasive diagnostic approach that analyzes blood or other bodily fluids to detect molecular alterations, ctDNA, CTCs, and cfDNA [93]. cfDNA and ctDNA are related but distinct entities. cfDNA represents the total pool of extracellular DNA fragments present in circulation and is derived from both malignant and non-malignant cells, whereas ctDNA represents the tumor-derived fraction of cfDNA and can be identified through multiple tumor-associated molecular features, including somatic genetic alterations, aberrant DNA methylation patterns and other epigenetic signatures, rather than exclusively through tumor-specific mutations [94,95]. While both tumor-informed and tumor-uninformed assays are being utilized in solid tumors such as non-small cell lung cancer, colon cancer and breast cancer, the role in HCC is still evolving [96]. Tumor-informed assays first characterize the patient’s tumor tissue and subsequently track selected patient-specific alterations in plasma, whereas tumor-uninformed, or tumor-agnostic, assays interrogate plasma directly using predefined genomic, epigenomic or other molecular signatures such as tumor-associated DNA methylation profiles, without requiring prior tumor sequencing [97]. Tumor-informed approaches may provide greater analytical sensitivity and specificity, particularly when ctDNA levels are very low, whereas tumor-uninformed approaches have the advantage of not requiring available tumor tissue, which may be particularly relevant in HCC where tissue acquisition is not always required for diagnosis [98].

#### 2.11.1. Circulating Tumor DNA and Cell-Free DNA

ctDNA is tumor-derived DNA released into circulation through cell death, including apoptosis and necrosis, and potentially through active release mechanisms [99]. In a narrative review summarizing comparative analyses, ctDNA outperformed AFP in both sensitivity (85%vs. 60%) and specificity (92% vs. 80%) in diagnostic accuracy for HCC detection [99]. cfDNA, commonly found in serum or plasma, typically consists of double-stranded DNA molecules. Currently, classical testing cannot distinguish ctDNA from other DNA types found in circulation. Only mutations specific to tumor cells on cfDNA can indicate its presence [100].

##### Advanced HCC Treated with ICI in a Single Cohort

A prospective study evaluating plasma biomarkers in HCC focused on cfDNA, ctDNA and cytokines in a cohort of 38 patients found that higher cfDNA and ctDNA levels both at baseline and at 3 months of therapy were associated with decreased survival and lack of radiological response to ICI therapy [101]. Patients with cfDNA levels more than 3.32 ng/μL at diagnosis had significantly higher mortality compared to those with levels below this threshold (median survival time 8 months versus 26 months respectively, *p* = 0.0001) [101]. A threshold of ctDNA 2.09 ng/μL was associated with a median survival time of 10 months for patients with levels above this threshold versus 24 months for patients with levels below this threshold (*p* = 0.0071), with 81.82% sensitivity and 87.5% specificity [101]. Similarly, higher baseline ctDNA levels were significantly associated with reduced OS (HR 1.080, CI 1.010–1.154, *p* = 0.024) and after 3 months of ICI (HR 1.079, 95% CI 1.003–1.161, *p* = 0.041). In relationship with radiological response, lower cfDNA and ctDNA levels had potential treatment-associated correlation with radiological response at baseline (cfDNA; CR 1.88 vs. PD 9.26 ng/μL, *p* = 0.0194, ctDNA; CR 0.99 vs. PD 5.68 ng/μL, *p* = 0.04) and at 3 months follow-up (cfDNA; CR 3.02 vs. PD 11.36 ng/μL, *p* = 0.026, ctDNA; CR 2.2 vs. PD 4.5 ng/μL, *p* = 0.046) respectively [101].

##### Prognostic Biomarker Post-Surgical Resection

A systematic review and meta-analysis of ctDNA in ten retrospective studies found that postoperative detectable ctDNA was associated with shorter relapse-free survival (RFS) (HR 4.48; 95% CI 2.56–7.82; *p* < 0.001) as well as worse OS (HR 2.99; 95% CI 1.94–4.61; *p* < 0.001) [102]. Baseline ctDNA detection was associated with reduced RFS (HR 3.54; 95% CI 1.97–6.38; *p*  <  0.001). They also found that sensitivity had a wide range of 33–82%, as well as specificity of 41–100%, highlighting the heterogeneity and non-uniformity of ctDNA in diagnostic performance [102]. An ongoing trial of ctDNA monitoring in patients with HCC and mCRC—NCT07001085—investigates the potential of ctDNA as a parameter for early detection of recurrence and prediction of therapeutic outcomes [103]. There are no current ongoing trials directly studying cfDNA, but there is a related trial evaluating cell-free tsRNA, small non-coding RNAs generated by the cleavage of transfer RNAs, for early detection of HCC [104]. More prospective trials incorporating ctDNA and cfDNA in HCC surveillance and guiding adjuvant treatment are still needed to allow wider adoption and standardization.

Detecting molecular residual disease (MRD) after curative-intent therapy demands very high analytical sensitivity and specificity, because residual ctDNA levels can be extremely low [102,105]. False-positive or false-negative results can substantially affect risk stratification. In contrast, patients with advanced HCC generally have a greater circulating tumor burden, making serial quantitative or molecular changes in ctDNA more readily detectable [105,106]. Accordingly, assays suitable for molecular profiling or response monitoring in advanced disease may not necessarily provide sufficient sensitivity for postoperative MRD. Clinical utility for treatment decisions based on MRD remains under investigation rather than established across solid tumors, including HCC.

Moving beyond traditional protein markers like AFP, liquid biopsies with ctDNA offer a non-invasive solution for real-time monitoring. This technology may eventually allow clinicians to use a more sensitive biomarker to track disease burden before, during, and after treatment, providing a more dynamic view of patient response than conventional diagnostics. Several analytical considerations may influence the performance and reproducibility of ctDNA-based biomarkers. The proportion of ctDNA within total cfDNA, commonly referred to as the tumor fraction, varies according to tumor burden, disease stage, tumor shedding, and treatment status, and a low tumor fraction represents an important cause of false-negative ctDNA results [105]. This is particularly relevant for early-stage disease and the post-treatment setting, where ctDNA may represent only a very small fraction of cfDNA and may fall below the analytical limit of detection. In HCC, underlying chronic liver disease may further complicate cfDNA interpretation because hepatocyte injury, inflammation, and cirrhosis can contribute non-tumor-derived DNA to the cfDNA pool [107,108,109]. Furthermore, preanalytical variables including blood collection tube type, time from collection to plasma separation, centrifugation conditions, plasma storage, freeze–thaw cycles, and DNA extraction methods may affect cfDNA yield and downstream ctDNA detection [110]. Standardization of these factors is therefore essential before ctDNA assays can be reliably incorporated into routine HCC management.

#### 2.11.2. Circulating Tumor Cells

CTCs are malignant cells that detach from a primary tumor and enter the circulatory system. They may disseminate through the bloodstream and contribute to distant metastases. CTCs generate valuable information about the genetics of the original tumors [99]. CTCs can potentially monitor disease progression, assess treatment efficacy, and detect early metastasis or recurrence [111]. The limitations in the clinical utility of CTCs are due to their extremely low numbers and variable persistence in the bloodstream. Improved detection techniques are needed for increased clinical utility of CTCs in comparison to ctDNA and cfDNA [99].

Nonetheless, one investigated subtype is EpCAM+ CTCs. A meta-analysis across solid tumors associated elevated tissue EpCAM expression with poorer prognosis; it did not specifically validate an EpCAM-enriched CTC assay [112]. However, only 20–52.5% of HCC tumors express EpCAM, which limits the applicability and clinical utility of this approach. A recognized limitation of EpCAM-dependent approaches in HCC is due to heterogeneous EpCAM expression and phenotypic changes. Therefore, EpCAM-independent approaches and regulatory mechanisms remain poorly understood [113]. There is also an ongoing clinical trial, NCT07303712, evaluating whether baseline CTC, cfDNA, and single circulating tumor cell-DNA (sCTC DNA) in the post-treatment period can be associated with treatment response to Y-90 in patients with HCC [114].

##### Summary

This review summarizes the growing research and exploratory data subsets suggesting the use of molecular and circulating biomarkers as potential prognostic biomarkers and predictors of clinical outcomes. Future randomized large-scale trials are essential to confirm their utility in clinical practice and guide individualized treatment decisions in HCC.

Table 1 highlights key studies evaluating these biomarkers, with a focus on clinical setting, disease stage, treatment modality, sample type and timing, utilized assays, results, prognostic versus predictive evidence and limitations.

## 3. Future Directions

Paired tissue analysis consisting of both normal tissue and tumor samples is needed to further characterize the treatment effects of immunotherapies. Also, further optimization of PD-L1 IHC testing and gene expression signature-based approaches are needed to allow for implementation into clinical practice given the current heterogeneity in these tests. Non-invasive liquid biopsy approaches will continue to explore ctDNA, cfDNA, and CTCs to improve the sensitivity and detection of these tests, and may ultimately become clinically applicable in HCC, as they have in other tumor types. Finally, AI-based approaches are emerging in HCC to analyze genomic data, develop risk prediction models and integrate multimodal data for precision oncology.

## 4. Conclusions

This review examines the distinctive TME of HCC and explores potential biomarkers investigated for their prognostic and potentially treatment-associated response to immunotherapy. Overall, the immune markers discussed in the review have shown correlation with prognosis and have suggested a possible treatment-related association role as well. PD-L1 and TMB have heterogeneous testing modalities and inconsistent association with ICI outcomes, but there is a trend for improved outcomes with ICIs. For immune cells, a high density of CD8^+^ T cells is associated with better PFS and OS, but resection-based prognosis, advanced-disease survival, and perioperative pharmacodynamic changes represent different endpoints and settings. Gene expression profiles, including inflammatory gene signatures, have been shown to correlate with outcomes in patients receiving ICIs. As for tumor intrinsic pathways, CTNNB1 mutation was suggested to be associated with less response to ICIs. Immune-related non-coding RNAs have been shown to be prognostic, but additional data are needed to evaluate their predictive role. NLR, PLR, AFP, microbiome features, and ctDNA may provide prognostic or monitoring information, but their associations are vulnerable to confounding and methodological heterogeneity. GALAD and BALAD-2 findings from screening or non-ICI cohorts should not be interpreted as evidence of immunotherapy selection. None of these exploratory markers are validated for routine selection among standard HCC ICI regimens.

As a narrative review, this manuscript did not employ a predefined systematic search strategy, formal study-selection criteria, or risk-of-bias assessment. Consequently, selection bias and incomplete literature ascertainment cannot be excluded, and the heterogeneity of included study designs should be considered when interpreting the available evidence. Current evidence and ongoing research efforts are limited by small studies and retrospective and exploratory data sets. Additionally, the heterogeneous evidence across studies is not unified to a single HCC clinical setting, operable or advanced, non-resectable disease. On the same level, the correlation investigated between systemic ICIs and biomarkers would require further validation stratified by type of immune therapy; single, dual checkpoint blockade, CTLA- or VEGF-based therapy. Another limitation of tissue-based biomarkers in HCC is the limited availability and representativeness of pretreatment tumor tissue, as HCC can frequently be diagnosed radiologically without histologic confirmation. Moreover, many available biomarker studies rely on resection specimens from early-stage disease or small biopsy samples that may not reflect the biology of advanced HCC treated with systemic immunotherapy. Spatial and temporal intratumoral heterogeneity, differences between primary and metastatic lesions, sampling error, and practical assay feasibility further limit reproducibility and remain important barriers to the routine clinical implementation of tissue-based immune biomarkers.

The underlying etiology of HCC contributes to heterogeneity within the TME, potentially influencing immune-cell infiltration, immune checkpoint expression and response to ICIs. Viral-associated HCC develops in the setting of chronic antigenic stimulation and persistent hepatic inflammation, promoting CD8^+^ T cells with overexpression of inhibitory receptors such as PD-1 [115,116]. These changes may influence PD-L1 expression and immune-related GEPs. Yet, consistent etiology-specific associated signatures have not been established [117,118,119]. Alcohol-associated liver disease promotes oxidative stress, gut-derived endotoxin exposure, and macrophage-mediated inflammation [120]. Collectively, they enhance immunosuppressive signaling and T-cell dysfunction. MASH-related HCC exhibits distinct immune alterations; e.g., accumulated yet dysfunctional CD8^+^ T cells, which may contribute to impaired antitumor immune surveillance, and potentially reduced efficacy of ICIs [121]. Although these etiological differences may influence the interpretation of TILs density, PD-L1 expression or GEPs, current evidence is insufficient to establish HCC-specific etiology or etiology-specific immune signatures as validated biomarkers for ICI selection [117,118,119]. Moreover, tumor extent and vascular invasion can influence AFP and ctDNA. Interpretation also requires separating tumor burden, hepatic reserve, and background inflammation. Hepatic reserve, assessed by Child–Pugh class, albumin–bilirubin (ALBI) grade, and prior or current decompensation, influences survival and treatment tolerance independently of tumor immunity [122]. Analyses should therefore adjust for these domains separately, alongside disease stage and treatment regimen. Exploratory associations with pre-existing immunity require additional standardization and validation before they can guide ICI treatment selection.

## Figures and Tables

**Table 1:** Select studies/trials evaluating potential circulating and immune biomarkers in HCC.

Study/Trial (Reference)	Clinical Setting	Treatment	Sample Type and Timing	Assay	Endpoint	Biomarker	Results	Prognostic and Predictive classification	Limitations
CheckMate 040 trial [26]	Advanced HCC	Nivolumab monotherapy	Pretreatment fresh, archival tumor and blood samples	(1) IHC: Dako PD-L1 IHC 28-8 pharmDx assay and T-cell/macrophage staining (2) RNA-sequencing: 4-gene inflammatory signature profiling (3) Central serologic testing, local complete blood counts	OS	PD-L1	Tumor PD-L1+ had better OS (median OS of 28.1 months vs. 16.6 months) (*p* = 0.032)	Prognostic	Design: Retrospective and single-arm (no control group), making findings prognostic rather than definitively predictive Sample Size: Constrained by limited baseline tissue availability
						CD3^+^, CD8^+^	Increased CD3^+^ and CD8^+^ TILs showed improved OS, although this was non- significant (*p* = 0.08)		
						GEP	CD274^+^ (PD-L1 messenger RNA), CD8A, LAG3, and STAT1 are associated with improved overall responses (*p* = 0.05) and OS (*p* = 0.01)		
						NLR	Lower NLR shows more response and longer OS than high NLR (*p* = 0.015)		
						PLR	Lower PLR shows more response to treatment. (*p* = 0.05)		
						AFP	AFP less than 400 μg/L has a higher OS		
Xue JS et al.; a systematic review and meta-analysis [32]	HCC prognostic evaluation after various treatments	Mixed treatments modalities including surgical resection, liver transplantation, TACE, ablation, radiation therapy, or multiple systemic therapies	Serum or plasma levels measured at baseline before treatment	ELISA in most studies, alongside antibody array assay and flow cytometric analysis	OS, DFS, RFS, TTP	sPD-L1	High sPD-L1 had worse OS (HR = 2.46, 95% CI 1.74–3.49, *p* < 0.001)	Prognostic	The reliability of these findings is weakened by significant inconsistencies across the included studies, particularly a lack of standardized measurement cut-offs, diverse patient backgrounds, and small sample sizes
						sPD-1	sPD-1 was not correlated with OS (HR = 1.19, 95% CI 0.55–2.56, *p* = 0.657)		
Xie, F., et al. [36]	HCC cohort from the Cancer Genome Atlas (TCGA)	Not explicitly focused on a single therapeutic Intervention (observational multi-omics analysis of genomic and immune profiles)	Bulk tumor tissue samples and RNA sequencing/somatic mutation data collected at baseline/diagnosis	NGS/RNA sequencing, VarScan 2.4.2 software for somatic mutations, and CIBERSORT algorithm	OS	TMB	High TMB associated with worse prognosis (*p* = 0.017)	Prognostic	These retrospective findings lack precise cellular resolution due to the use of bulk sequencing, and they carry a selection bias favoring patients with well-preserved liver function
GO30140 (Phase 1b) [20]	Unresectable HCC in patients who had no previous systemic therapy	Atezolizumab + bevacizumab, or atezolizumab monotherapy	Baseline tumor tissues (blood-matched samples for whole-exome sequencing), and paired pre- and post-treatment biopsies	Transcriptome analysis (RNA sequencing), whole-exome sequencing (WES), FoundationOne panel profiling, IHC, and multiplex IHC with digital pathology	ORR PFS OS	TMB	ORR was higher in high TMB (56%) than in low (35%) or medium TMB (17%), and high TMB showed a nonsignificant trend of longer PFS compared to low and medium TMB	Prognostic	Exploratory nature of several subsets and the reliability of these findings is limited by post hoc tissue sampling bias, retrospectively defined biomarker cut-offs that require prospective validation
						CD8^+^, CD3^+^ and GZMB^+^CD3^+^	Responders had higher density of CD8^+^ T cells, CD3^+^ T cells and GZMB^+^CD3^+^ T cells than non-responders		
IMbrave150 (Phase 3 randomized trial) [20]	Unresectable HCC in patients who had no previous systemic therapy	Atezolizumab + bevacizumab combination versus sorafenib	Baseline tumor tissues (blood-matched samples for whole-exome sequencing), and paired pre- and post-treatment biopsies	Transcriptome analysis (RNA sequencing), whole-exome sequencing (WES), FoundationOne panel profiling, IHC, and multiplex IHC with digital pathology	ORR PFS OS	TMB	ORR of 37% in the TMB-high group and 38% in the TMB-low group.	Potential predictive	Exploratory nature of several subsets and the reliability of these findings is limited by post hoc tissue sampling bias, retrospectively defined biomarker cut-offs that require prospective validation
						CD8^+^, CD3^+^ and GZMB^+^CD3^+^	Responders had a higher density of CD8^+^ T cells, CD3^+^ T cells and GZMB^+^CD3^+^ T cells than non-responders		
						GEP	Patients with high ABRS (treatment-specific gene signature), high CD274^+^, and high T-effector signatures experienced a significant PFS benefit (*p* = 0.013)		
						Tumor-Intrinsic Pathways	CTNNB1 wild-type and TERT mutant tumors were associated with greater benefits with atezolizumab and bevacizumab compared to sorafenib		
Gao, F., et al. A two-phase correlative study [45]	Two HCC cohorts; discovery and validation phases	Surgically resected HCC without pretreatment	Formalin-fixed, paraffin-embedded HCC tumor and adjacent tissues evaluated at baseline/surgery using H&E-stained sections	Histopathologic evaluation using the International TILs Working Group criteria on H&E-stained sections, IHC against CD8^+^, CD68^+^, CD4^+^, FOXP3, PD-1, PD-L1, and OX40	OS DFS	TIL	High (TILs ≥ 50%) and intermediate (10% ≤ TILs < 50%) TIL groups had better OS and DFS compared to the low (TILs < 10%) TIL group	Prognostic	The retrospective design across distinct cohorts supports generalizability, but the low prevalence of high-TIL tumors limits the statistical power to definitively evaluate that specific subgroup
Duffy, G.A., et al. NCT01853618 [46]	Advanced HCC	Tremelimumab combined with subtotal radiofrequency ablation, chemoablation or TACE	(1) Peripheral blood mononuclear cells collected every cycle (2) Optional 6-week post-treatment tumor biopsies compared to pre-treatment archival samples (3) Blood and viral load monitoring every 4 weeks	(1) Multicolor flow cytometry for peripheral T cell subsets (2) IHC with automated image analysis for CD3^+^ and CD8^+^ in tumor biopsies (3) Standard quantitative PCR and EIA assays for viral load and hepatitis markers	Feasibility of ICI ORR TTP PFS OS	CD3^+^ and CD8^+^	Responders had higher CD3^+^ and CD8^+^ T cells (10.21% and 8.1%, respectively) compared to non-responders (2.44% and 2.00% respectively)	Prognostic	The small, non-randomized pilot design prevents isolating the specific therapeutic effect of tremelimumab from the concurrent ablation or TACE procedures
Lapelusa, M., et al. Correlative analysis of phase II trial NCT03222076 [28]	Early stage, resectable HCC	Neoadjuvant immunotherapy using nivolumab plus ipilimumab or nivolumab alone, followed by surgical resection	(1) Serial tissue biopsies obtained pretreatment and after completing neoadjuvant immunotherapy (2) Surgical resection specimens (for pathologic response evaluation)	IHC with Halo image analysis software v3.2 (Indica Labs, NM, USA) for quantitative assessment of CD8^+^, Gr-B, PD-1, and PD-L1 quantification	MPR ORR TTP PFS	CD8^+^	Patients with MPR (>70% tumor necrosis) had greater increases in intratumoral CD8^+^ expression compared to non-responders (26.92% vs. -0.04%; *p* = 0.026)	Prognostic and exploratory treatment-associated response	The findings are primarily hypothesis-generating due to a small, single-institution cohort (N = 18) and potential evaluation biases stemming from spatially limited core biopsies and unblinded radiological assessments
						PD-1/PD-L1	Patients with MPR had a greater increase in intratumoral expression levels of PD-1 (20.17% vs. 0.40%; *p* = 0.048) but not PD-L1 (7.69% vs. 0.57%; *p* = 0.26)		
Peng, L., et al. [55]	33 types of cancers including HCC	ICI therapy targeting the PD-1/PD-L1 axis and CTLA-4 (evaluated via prognostic and correlative biomarker analysis)	(1) Bioinformatics cohorts from TCGA and GTEx databases (2) Clinical frozen resected tumor tissue specimens	(1) Computational bioinformatic analyses via TIMER (https://cistrome.shinyapps.io/timer/), GEPIA (http://gepia.cancer-pku.cn/), and Cytoscape (https://cytoscape.org/ ) (ClueGO/CluePedia for GO and KEGG pathway enrichment) (2) Quantitative real-time reverse transcription PCR for clinical specimen validation	OS DFS	lncRNAs	High expression of the lncRNAs in the MIR155 host gene was significantly correlated with infiltrating immune cells as well as with PD-L1 and CTLA4	Prognostic	The findings rely heavily on public databases and a small validation cohort, meaning further experimental research is needed to confirm the exact molecular mechanisms of MIR155HG
Mao, J. et al. [91]	65 patients with advanced HCC and biliary tract cancer (30:35 ratio)	Anti-PD-1 ICI after progression on 1st line chemotherapy (Gemcitabine + Cisplatin)	Fresh stool samples were collected prospectively before and during anti-PD-1 treatment every 3 weeks	Metagenomic sequencing of fresh stool samples using the Illumina NovaSeq 6000 platform, followed by taxonomic profiling (Kraken 2) and functional annotation (KEGG and MetaCyc databases)	Differentiation of microbial enriched taxa correlated with radiological response according to RECIST.1.1	Gut Microbiome	A high abundance of *Lachnospiraceae bacterium* GAM79 and *Alistipes* sp. Marseille-P5997 correlated with significantly longer OS (*p* = 0.023 & *p* = 0.021 respectively) and improved PFS (*p* = 0.020 & 0.013 respectively)	Prognostic	The reliability of these findings is constrained by a small, heterogeneous, mixed cohort of hepatobiliary cancer, unmeasured clinical confounders like diet and antibiotics, and a lack of mechanistic in vivo validation to prove causality
Chung, M.-W., et al. [92]	Advanced HCC, who received nivolumab after sorafenib failure	Nivolumab administered as a second- or third-line systemic immunotherapy	Fecal samples collected prospectively at baseline <1 month before the first nivolumab injection and at 3 months follow-up if progressive disease and at 5–7 months if responded to nivolumab	16S ribosomal RNA gene sequencing targeting the V3-V4 region using the Illumina MiSeq platform, with metagenomic data processed via CLC Genomics Workbench and the FROGS guidelines	Metagenomic and microbiome data from 16S ribosomal RNA sequencing analyzed according to therapeutic response	Gut Microbiome	Responders to nivolumab demonstrated a higher Shannon index, a measure of microbial diversity reflecting both species’ richness and evenness, indicating significantly greater bacterial richness and diversity compared to non-responders (*p* = 0.036)	Prognostic	These findings are limited in statistical power and generalizability by a very small, single-center cohort (N = 8) and potential individual confounding factors
Vargas-Accarino, E., et al. [101]	Advanced HCC treated with ICIs	ICIs, including nivolumab, atezolizumab/bevacizumab, durvalumab/tremelimumab, lenvatinib/pembrolizumab, and pembrolizumab; 44.7% of patients had previous TKIs	Plasma samples collected prospectively at baseline and three months post-treatment initiation	(1) Droplet digital PCR for TERT promoter mutations (2) Onco-500 TruSight panel for ctDNA genomic profiling	Measure plasma levels of cfDNA, ctDNA and cytokines and assess correlation with response to ICIs	cfDNA	Higher cfDNA levels at diagnosis were associated with increased mortality during follow-up (*p* = 0.0001), and lower levels at baseline and after three months of ICI correlated with radiological response (*p* = 0.0194, *p* = 0.026 respectively)	Prognostic	Small sample size (N = 38 patients) and single-center study design, requiring validation in larger multi-institutional cohorts
						ctDNA	Higher ctDNA levels were associated with reduced OS at baseline (HR 1.080, *p* = 0.024) and 3 months following ICI (HR 1.079, *p* = 0.041); lower levels at baseline and after three months of ICI correlated with radiological response (*p* = 0.04, *p* = 0.046 respectively)		
Buonopane IR et al. a systematic review and meta-analysis [102]	Non-metastatic HCC undergoing curative-intent treatment	Curative-intent treatments among 793 patients, including surgical resection (474 patients), liver transplantation (163 patients), and percutaneous ablation (103 patients)	ctDNA was obtained exclusively in the postoperative setting in 7 out of 10 studies, using variable methods of ctDNA detection	NGS was used in 7 studies and NGS combined with PCR was used in 2 studies	RFS OS	ctDNA	Postoperative ctDNA detection was associated with shorter RFS (HR 4.48; 95% CI 2.56–7.82; *p* < 0.001 as well as worse OS (HR 2.99; 95% CI 1.94–4.61; *p* < 0.001)	Prognostic	The reliability of these findings is limited by the inclusion of low-quality retrospective studies, potential publication bias, and highly variable ctDNA testing methods (assays, cut-offs, and timing)

This table represents select studies evaluating circulating and potential biomarkers in HCC to summarize results, evidence classification and limitations within various clinical settings, therapeutics, tissue or blood samples and assays. This table does not serve as a comprehensive presentation of all biomarkers or studies. Abbreviations: HCC, hepatocellular carcinoma; PD-L1, programmed death ligand-1; OS, overall survival; TIL; tumor-infiltrating lymphocytes; NLR, neutrophil-to-lymphocyte ratio; PLR, platelet-to-lymphocyte ratio; AFP, alpha-fetoprotein; GEP, gene expression profiles; sPD-L1, soluble programmed death ligand-1, sPD-1, soluble programmed death-1; TACE, transarterial chemoembolization; DFS; disease-free survival; RFS; relapse-free survival; TTP; time to tumor progression; TMB, tumor mutation burden; ORR, objective response rate; PFS, progression-free survival; MPR, major pathologic response; ICI, immune-checkpoint inhibitor; lncRNA, long non-coding RNAs; CTLA4, cytotoxic T-lymphocyte-associated protein 4; TKI; tyrosine-kinase inhibitor; cfDNA, cell-free DNA; ctDNA, circulating-tumor DNA; IHC, immunohistochemistry; ELISA, enzyme-linked immunosorbent assay; NGS, next-generation sequencing; H&E, Hematoxylin and eosin; PCR, polymerase-chain reaction; EIA, enzyme immunoassay; GEPIA, Gene Expression Profiling Interactive Analysis; GO, Gene Ontology; TIMER, Tumor Immune Estimation Resource; KEGG, Kyoto Encyclopedia of Genes and Genomes.

## Data Availability

No new data were created or analyzed in this study. Data sharing is not applicable to this article.

## Abbreviations

Hepatocellular carcinoma (HCC), vascular endothelial growth factor (VEGF), immune checkpoint inhibitors (ICI), tyrosine-kinase inhibitors (TKIs), programmed death-ligand 1 (PD-L1), overall survival (OS), objective response rates (ORR), cytotoxic T-lymphocyte-associated protein 4 (CTLA-4), major histocompatibility complex class I (MHC-I), tumor microenvironment (TME), regulatory T-cells (Tregs), myeloid-derived suppressor cells (MDSCs), tumor-associated macrophages (TAMs), alcohol-associated liver disease (ALD), metabolic dysfunction-associated steatohepatitis (MASH), interferon gamma (IFN-γ), transforming growth factor beta (TGF-β), tumor infiltrating lymphocytes (TILs), long non-coding RNAs (lncRNAs), tumor mutational burden (TMB), soluble PD-L1 (sPD-L1), circulating tumor DNA (ctDNA), cell-free DNA (cfDNA), circulating tumor cells (CTCs), alpha-fetoprotein (AFP), complete response (CR), partial response (PR), immunohistochemistry (IHC), major pathological response (MPR), soluble PD-1 (sPD-1), disease-free survival (DFS), relapse-free survival (RFS), time to progression (TTP), tumor-free survival (TFS), mutations per megabase (mut/Mb), the cancer genome atlas (TCGA), human telomerase reverse transcriptase (hTERT), next-generation sequencing (NGS), stable disease (SD), progressive disease (PD), mean positive pixel count (mPPC), artificial intelligence (AI), automated CD8^+^ tumor-infiltrating lymphocyte scoring system (ATLS-8), tumor endothelial cell (TEC), Gene expression profiles (GEPs), disease control rate (DCR), neutrophil-to-lymphocyte ratio (NLR), platelet-to-lymphocyte ratio (PLR), transarterial chemoembolization (TACE), national liver cancer screening trial (TRACER), lens culinaris agglutinin-reactive AFP (AFP-L3), des-gamma-carboxy prothrombin (DCP), microbiota-associated molecular patterns (MAMPs), lipopolysaccharide (LPS), hepatic stellate cells (HSCs), Toll-like receptor 4 (TLR4), deoxycholic acid (DCA), fecal microbiota transplantation (FMT), interleukin-12 (IL-12), molecular residual disease (MRD), albumin–bilirubin (ALBI), Barcelona clinic liver cancer (BCLC), hematoxylin and eosin (H&E), modified response evaluation criteria in solid tumors (mRECIST), single circulating tumor cell-DNA (sCTC DNA).
